# Interception by two predatory fly species is explained by a proportional navigation feedback controller

**DOI:** 10.1098/rsif.2018.0466

**Published:** 2018-10-17

**Authors:** Samuel T. Fabian, Mary E. Sumner, Trevor J. Wardill, Sergio Rossoni, Paloma T. Gonzalez-Bellido

**Affiliations:** 1 Department of Physiology, Development and Neuroscience, University of Cambridge, Downing Street, Cambridge CB3 2EG, UK; 2 Department of Ecology, Evolution, and Behavior, University of Minnesota, Saint Paul, MN 55108, USA

**Keywords:** predation, interception, flight, control system, insect

## Abstract

When aiming to capture a fast-moving target, animals can follow it until they catch up, or try to intercept it. In principle, interception is the more complicated strategy, but also more energy efficient. To study whether simple feedback controllers can explain interception behaviours by animals with miniature brains, we have reconstructed and studied the predatory flights of the robber fly *Holcocephala fusca* and killer fly *Coenosia attenuata*. Although both species catch other aerial arthropods out of the air, *Holcocephala* contrasts prey against the open sky, while *Coenosia* hunts against clutter and at much closer range. Thus, their solutions to this target catching task may differ significantly. We reconstructed in three dimensions the flight trajectories of these two species and those of the presented targets they were attempting to intercept. We then tested their recorded performances against simulations. We found that both species intercept targets on near time-optimal courses. To investigate the guidance laws that could underlie this behaviour, we tested three alternative control systems (pure pursuit, deviated pursuit and proportional navigation). Only proportional navigation explains the timing and magnitude of fly steering responses, but with differing gain constants and delays for each fly species. *Holcocephala* uses a dimensionless navigational constant of *N* ≈ 3 with a time delay of ≈28 ms to intercept targets over a comparatively long range. This constant is optimal, as it minimizes the control effort required to hit the target. In contrast, *Coenosia* uses a constant of *N* ≈ 1.5 with a time delay of ≈18 ms, this setting may allow *Coenosia* to cope with the extremely high line-of-sight rotation rates, which are due to close target proximity, and thus prevent overcompensation of steering. This is the first clear evidence of interception supported by proportional navigation in insects. This work also demonstrates how by setting different gains and delays, the same simple feedback controller can yield the necessary performance in two different environments.

## 1. Introduction

After detecting a moving object, animals must choose the appropriate response. Irrelevant objects ought to be ignored [1] and escape or freeze responses used to avoid predation [2]. In contrast, a potential mate or prey on the move should be approached or followed using a swift and precise tactic, such as pursuit or interception [3–5]. This high performance behaviour is common in aerial [3,6], terrestrial [7] and aquatic habitats [8,9], and is conducted by species widely distributed across the phylogenetic tree, from small insects to large mammals. Nevertheless, all chasers face the need to maximize the probability of success and minimize effort expended. The task of catching a moving object can be solved, broadly, by two tactics. A chaser can navigate so that (i) it heads directly for the perceived location of the target, a strategy known as pursuit, or (ii) it moves towards a point ahead of the target's current location, called interception. Pursuit navigation towards moving targets has been reported in houseflies [4,10], tiger beetles [7], honeybees [11] and long legged flies [12]. In contrast, species that intercept their targets include bats [13], hawks [14], falcons [15], hoverflies [3], dragonflies [6], miniature robber flies [16] and humans [17].

**Figure 1.**
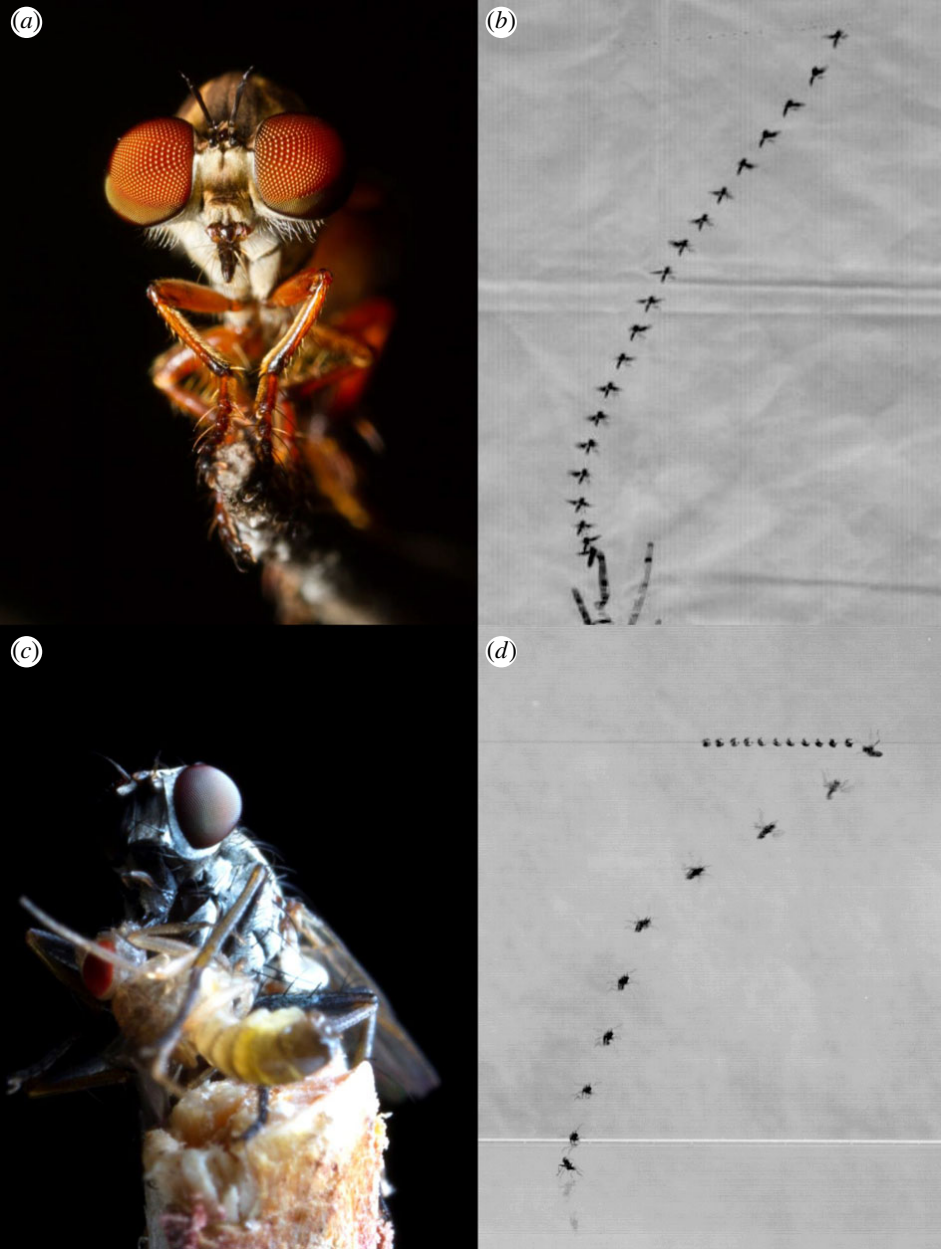
Predatory attack of two miniature dipteran species. (*a*) *Holcocephala fusca* perched. (*b*) An overlay image of *Holcocephala* intercepting a dummy target. (*c*) *Coenosia attenuata* with a fruit fly previously caught mid-flight. (*d*) An overlay image of *Coenosia* intercepting a dummy target.

Through interception, a chaser can reach the target faster than if using pursuit, but in principle interception is a more difficult strategy. To take an optimal course, the chaser must gauge how far ahead of a target to aim, a product of the velocity of both pursuer and prey. However, since miniature dipteran flies (i.e. *Holcocephala fusca* [16]) with limited neural capacity can intercept their target, a relatively simple and robust guidance law is likely in place in some animals. Indeed, interception can be driven by a simple feedback controller, and this type of controller explains the final approach in predatory flights of falcons, birds of prey with complex brains [15]. In addition to a feedback controller, dragonflies are believed to use a predictive mechanism to reduce lag in head-tracking the target, i.e. using internal models of both their own bodies and prey flightpaths, something that could be used to aid steering an interception course [18]. Small predatory flies tackle what is essentially the same predatory task as that of dragonflies. Have their miniature brains also evolved a predictive system? Or are feedback controllers tuned to the requirements of the species sufficient? Here, we investigate if a feedback controller can explain the aerial hunts of a North American robber fly (figure 1*a*) (*Holcocephala fusca*, 6 mm body size) and a Mediterranean killer fly (figure 1*c*) (*Coenosia attenuata,* 4 mm body size) towards objects moving with either constant or erratic velocities (example behaviours in figure 1*b* for *Holcocephala* and figure 1*c* for *Coenosia*). Both of these miniature dipteran species are sit-and-wait generalist predators [19,20] that catch their prey in mid-air. However, the two species differ in visual acuity (i.e. *Holcocephala* having an acute region with peak resolution 10× better that of *Coenosia* [16,21]) and environment (i.e. *Holcocephala* hunts against clear sky, while *Coenosia* can hunt against and between foliage). We have analysed their predatory flights and tested if these can be predicted with any of the three most probable simple controllers; (i) pure pursuit, (ii) deviated pursuit or (iii) proportional navigation. These feedback controllers apply changes to the heading of the pursuer (equivalent to the direction of the pursuer velocity vector) corresponding to different stimuli.

**Figure 2.**
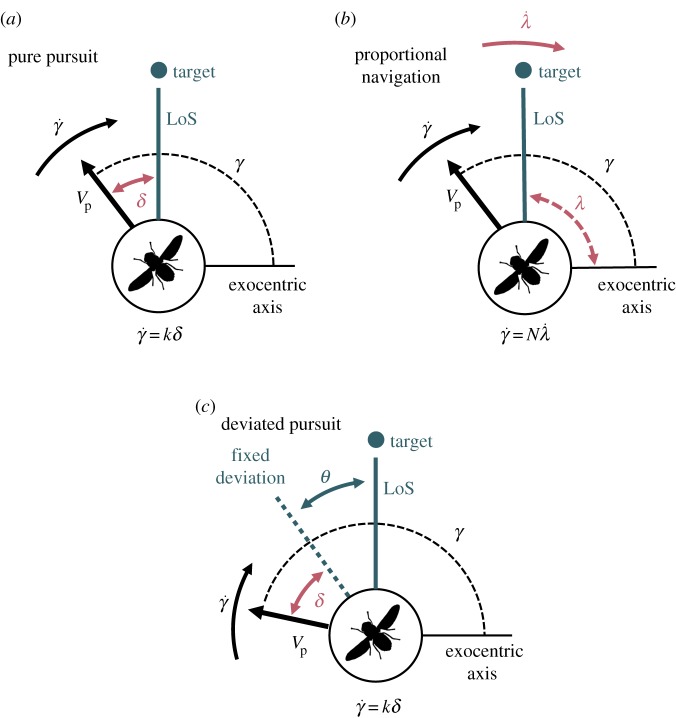
Diagrammatic representations of a pure pursuit and proportional navigation controller. (*a*) Under a pure pursuit controller, the error (*δ*) between pursuer's heading (equivalent to direction of velocity vector) (*V*_p_) and line-of-sight (LoS) to the target is minimized through correctional rotation of the heading that alters the path angle (*γ*). The turns are in proportion to the perceived error, with *k* as the constant of proportionality. (*b*) Under a proportional navigation controller, it is rotation in the LoS that stimulates rotation of the pursuer heading (*V*_p_) about the same axis, magnified by a navigation constant *N*. Rotation of the LoS is measured by the angle (*λ*) made between LoS and an exocentric reference frame. (*c*) Under a deviated pursuit controller, the error (*δ*) value is taken between a fixed deviation (*θ*) from the LoS and the pursuer's heading (*V*_p_). Corrective turns are in proportion to this error as in a pure pursuit controller, with *k* as the constant of proportionality.

For *pure pursuit* (figure 2*a*), a controller measures an error angle (*δ*) between the pursuer heading (*V*_p_) and the line formed between pursuer and target, called line-of-sight (LoS). The controller then aims to minimize this error (*δ*) by steering to rotate the pursuer heading (*V*_p_). The strength of steering corrections applied (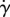) being relative to the magnitude of the initial error angle (*δ*) and the gain given by an intrinsic constant (*k*), as in the equation below.
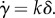

A *deviated pursuit* controller can shorten the pursuer trajectory by using the same control system as above but fixing the intended error angle (*δ*) at a positive value (instead of zero) [22]. This strategy is suitable for animals that can estimate absolute target size, or have this knowledge available innately, and thus can calculate the optimal error angle based on the angular size and speed of the target. This is the case for male hoverflies chasing females [3], but it would seem unsuitable for the two flies under investigation in this study, as they are generalist aerial predators and such size assumptions will not match to all potential prey items (although see [23] and [24] about heuristic target size assessment).

*Proportional navigation* (pro-nav) enables interception through a different feedback mechanism (figure 2*b*), and is the basis of navigation in most modern missiles [22]. A pro-nav controller aims to keep the direction of LoS constant across the trajectory, and by minimizing the LoS rate of rotation (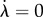). As a result, at any point during the trajectory, the rotation rate of the pursuer's heading (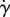) is proportional to 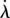, magnified by a gain set by the navigation constant (*N*), as in the equation below:
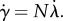


Proportional navigation simplifies the problem for intercepting targets of unknown absolute size and distance, as only the rotation rate of the LoS needs to be evaluated to ascertain whether the pursuer is going to collide with the target. The rotation rate is relative to an exocentric reference frame, achievable by summing together the visually perceived LoS rotation and known self-rotation. Therefore, pro-nav relies on the principle of parallel navigation [22], which states that if the LoS does not rotate relative to an exocentric reference frame (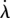) as the range between the target and pursuer closes, they are on a collision course. Bats [13], goshawks [14] and *Holcocephala* [16] have all been shown to be using the parallel navigation rule to intercept targets (although given other names: constant absolute target direction for both bats and hawks, constant bearing for *Holcocephala*). Importantly, parallel navigation describes a condition to be sought (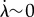), but it does not specify how this condition is achieved. Pro-nav serves as a parsimonious method to fulfil parallel navigation within human engineered systems [22]. While pro-nav has been implemented within modern missile guidance for many decades, its use by animals has been demonstrated only recently by Brighton *et al.* [15], on the terminal attack phase of aerial assaults by peregrine falcons. It should be noted that a pro-nav controller behaves like a deviated pursuit controller when the gain *N* = 1, as it maintains a fixed initial error angle, however the rates of pursuer turn are proportional to different stimuli. For further description of parallel navigation and proportional navigation, see electronic supplementary material 1.

Here, we have analysed and modelled the attacks of *Holcocephala* and *Coenosia* flights. The results are consistent with the use of a proportional navigation controller, with gain and delay adjusted to suit the adaptations of each species and the environments in which they operate.

## 2. Method

### 2.1. Animals and experiments

*Holcocephala fusca* used in experiments were from wild population and left *in situ*, experimental apparatus being arranged around their chosen perches. *Coenosia attenuata* were obtained either from lab-reared stocks of a captive population held within the University of Cambridge for experiments involving dummy targets, or from a wild population and left *in situ* in the greenhouse when intercepting ‘natural’ targets. Dummy target experiments conducted with *Coenosia* were in laboratory conditions and under artificial lighting (6.7 KLx upward, 1.2 KLx reflected).

Behavioural data were acquired with a pair of time-synchronized Photron Fastcam SA2's with overlapping fields of view so that three-dimensional placement of pursuer and target could be attained. Cameras were calibrated with known-sized checker boards. All flights were captured at 1000 frames per second and the placement of pursuer and target in both frames tracked. Temporal resolution of 1 ms is retained throughout all analysis. Raw positional data were smoothed to account for erroneous small perturbations generated through tracking that could lead to false measurement of LoS rotation. Further details given in supplementary information of [16] and for details on trajectory smoothing, see [25].

### 2.2. Visual stimulus

To elicit predatory behaviour, flies were presented with dummy targets. These targets took the form of silvered beads of variable diameters (1.3, 2.9 and 3.9 mm) on fishing line. To get the targets to pass in a straight line and at set speeds, the fishing line was passed around a U-frame with wheels at all four corners and a central stepper motor that controlled bead movement (see [16]). To move targets erratically for *Holcocephala*, and thus generate unpredictable changes in the LoS to the target, a single target was hung from a length of fishing line tied to a 30 cm long thin wooden stick. These targets could then be moved by an operator in front of the flies and produce variable, nonlinear trajectories where both the bearing and speed of the target varied greatly. To move targets erratically for *Coenosia*, fruit flies (*Drosophila melanogaster*) were released from a vial near a perched *Coenosia*.

Linear *Holcocephala* targets were tested across a speed range of 0.03–1.05 ms^−1^, a mean speed variation within flights of 0.12 ms^−1^ ± s.e. 0.01 ms^−1^ (*n* = 109) and a mean target heading deviation from initial conditions of 18.8° ± s.e. 3.0° (*n* = 109) within flights. For *Coenosia*, linear targets had a speed between 0.10–0.79 ms^−1^, a mean speed variation of 0.01 ms^−1^ ± s.e. 0.00 ms^−1^ (*n* = 59) within flights and a mean target heading deviation of 0.9° ± s.e. 0.1° (*n* = 59) within flights. *Holcocephala* erratic targets travelled between 0.07–0.73 ms^−1^, had a mean variation in speed of 0.42 ms^−1^ ± s.e. 0.05 ms^−1^ (*n* = 17) within flights and heading deviation of 62.4° ± s.e. 7.9° (*n* = 17) within flights. *Coenosia* erratic targets had a speed between 0.54–1.23 ms^−1^, an average variation in speed of 0.26 ms^−1^ ± s.e. 0.10 ms^−1^ (*n* = 8) within flights and a mean heading deviation of 35.4° ± s.e. 8.5° (*n* = 8) within flights.

### 2.3. Data analysis

All analysis of captured data was conducted in MATLAB 2016b with custom written scripts.

Only flights where contact with the target was made were included in the analysis. This is because in the attacks that were abandoned before the target was caught (*Coenosia* 43% and *Holcocephala* 36%), it was not clear at which point the flight motivation switched from interception to avoidance. The early part of the pursuer's trajectory, at the beginning of the hunt, was not included in the analysis as this reflected take-off dynamics (e.g. high accelerations). Hence, when models were being tested and applied, the start point was taken at 20% of the way through the flight course. Likewise, analysis was stopped 1 cm before the pursuer hit the target, as the final approach is dominated by ballistic movements. Individuals could not be tagged due to limitations related to field-based research and animal size. Hence, some flights may be from the same individuals. For *Holcocephala*, the flight data are from female and male flies, although the sex of the subject was not noted for each flight. For *Coenosia*, the flight data are from females. Male *Coenosia* were not used in this study as they are far less abundant in the colony than females and frequently take-off even when targets are not being presented.

To test for parallel navigation, range vector correlation is used as a measure of LoS parallelism, a measure that ties in with existing work (i.e. [16,18]) (see electronic supplementary material 1c.). The correlation value is given by (i) the angle difference between successive LoS vectors and (ii) the difference of their magnitudes. A value of 1 indicates that LoS are parallel and getting longer (i.e. the target is increasing range from pursuer), a value of 0 means that distance to the target is maintained but LoS are rotating and a value of −1 indicates that LoS are parallel and the range to the target is decreasing (i.e. perfect parallel navigation). Flight time was normalized to a percentage of flight complete so that all flights could be overlaid.

Optimum heading analysis was conducted by taking the properties of fly speed and the three components of motion of the target and solving a pair of simultaneous equations to distribute fly speed into the three components such that it will meet the target in X, Y and Z at the same time, signalling connection with the target. The equations are as follows:
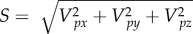

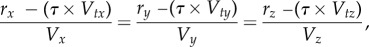
where *S* is pursuer speed. *V_px_*, *V_py_* and *V_pz_* are pursuer velocity components in three dimensions, of unknown magnitude. *r_x_*, *r_y_* and *r_z_* are the range vectors between target and pursuer. *V_tx_*, *V_ty_* and *V_tz_* are target velocity components of known magnitude. *τ* is an unknown time-to-contact.

Analysis was conducted in the full three-dimensions recorded, except for investigation into correlation between the rotation of LoS and pursuer velocity. In this case, flights were flattened to a two-dimensional engagement plane. Flattening to two-dimensions allows a polarity to be given to the rotation of both LoS and pursuer heading. This plane was defined by three points, the starting positions of fly and target and the greatest displacement of target from its origin. This gave the least amount of information loss about the flight course when the third dimension was removed (electronic supplementary material 2).

### 2.4. Feedback controllers and flight simulations

We simulated three models on recorded successful flights: pure pursuit, deviated pursuit and proportional navigation. These models are described in the introduction. To run the simulation, the forward speed of the predator during the actual flight was fed into the simulation. A fixed time delay, acquired from correlations in the entire dataset of each species, was also fed into the model. The temporal resolution of the models was equal to that of the flight (1 ms), which is well below the predicted reaction time of these flies (between 10 and 30 ms). These simulations then output the rotation in heading according either to (i) error between LoS and fly heading (Pure Pursuit) or (ii) rotation of LoS (pro-nav and *N* = 1 proxy for fixed angle of deviated pursuit). The simulations started at the same position and with the same heading as the fly at the beginning of the navigation phase of the flight, but after this point the LoS rate and error angle were measured with regards to the model alone, and not taken from the true flight.

The measure of fit between simulations and true trajectories was an angular error between the heading of the model and the heading of the true fly trajectory. To independently test which gain (navigational constant) provided the best fit, we sequentially fitted gains across a range (from 1 to 10, incrementing by 0.1). We define the best fitting gain as the one that resulted in the lowest mean error across the navigation phase of the flight. We used this method (instead of a distance measure between fly and model at all time points), because it provides a metric for how well the simulation matched the shape of actual pursuer trajectory, even if the position at which it did so differed from the real position of the fly. Time delays used in model fitting were taken from best fitting correlations of LoS rotation and pursuer velocity rotation (28 ms for *Holcocephala* and 18 ms for *Coenosia*). Pro-nav models are depicted with an arbitrary ±30% of fitted gain to demonstrate the sensitivity of flightpath to the chosen gain. Pure pursuit models are depicted with a 10× gain range from 10 to 100 s^−1^ to demonstrate a wide range of gains do not improve model fit. This range was chosen to encompass the gains described in the aerial pursuit of other insects [4,12].

We also tested for the advantage of using a pro-nav controller (tuned to either of the two fly species), versus a pure pursuit controller by carrying out flight simulations. Advantage was quantified as the percentage difference in time-to-contact between a pure pursuit and pro-nav. The difference was tested at different target speeds but with the same starting positions and headings. For the pure pursuit simulation, we used the mean starting positions for each species (acquired from the data) and set the trajectory starting from the pursuer's origin. For the pursuit course simulation, when flight-time exceeded that of the true flightpath, and the fly had not reached the target, target velocity and fly speed were extrapolated from the last available values. Similarly, a separate pro-nav model was also set off from the origin to test whether it had a similar advantage, over the pure pursuit model, as the true flights. The navigational constants for the pro-nav controllers (*N* = 3 for *Holcocephala* and *N* = 1.5 for *Coenosia*) in the simulations were taken from the best fitting correlative data in this study. The pursuit model took the constant value (*k* = 20 s^−1^) from recorded work for the housefly *Fannia canicularis* [4].

### 2.5. Tests for optimal take-off distance

We tested whether the timing of the predatory fly at take-off was time-optimal (i.e. whether it allowed the animal to intercept prey in the shortest possible time). For this analysis targets were simulated travelling left to right. Target movement was presented across a range of increasing altitude, spanning both approaching and receding distances. Targets were presented above a pursuer that sets off vertically from the origin. The pursuer is steered by a pro-nav model with its navigation constant matched with that particular to each species (for *Holcocephala N* = 3, delay = 28 ms, for *Coenosia N* = 1.5, delay = 18 ms). The speeds for target and pursuer used in the simulations were taken from the means from the recorded flight data for each species. Time-to-contact was measured and normalized for each target altitude. This allowed us to find the time/location along the target's horizontal flightpath where the fly should take-off to produce minimal time-to-contact. To compare the timing of the simulated (optimal) predatory take-off versus the timing of the real take-off, a common reference frame was necessary. To obtain it, the recorded flights were rotated until the linear target trajectories were aligned to the horizontal axis. We then noted the position of the target as the pursuer took off.

## 3. Results

### 3.1. Flight parameters

**Figure 3.**
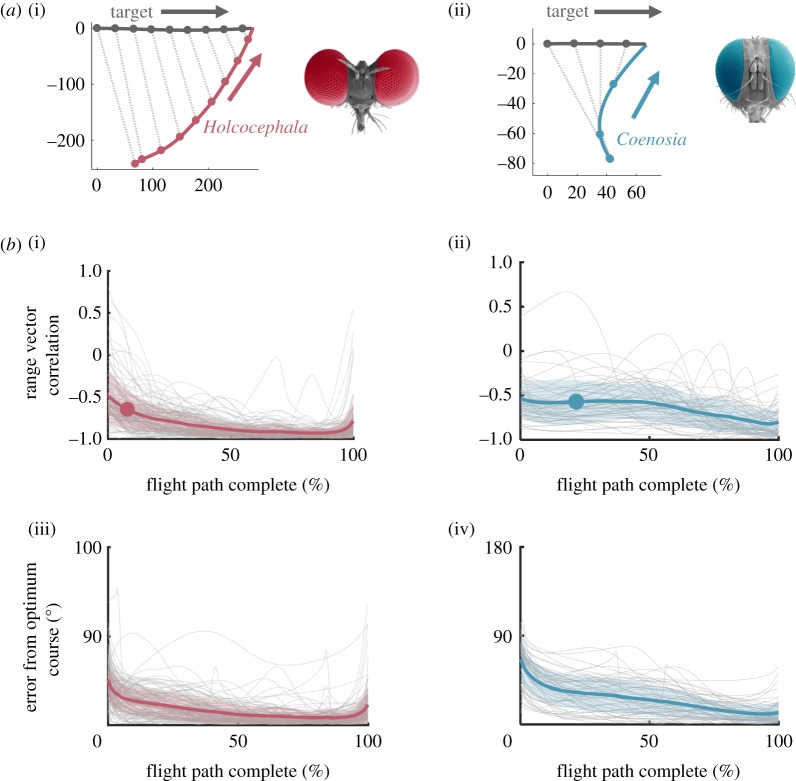
Flight profiles (*a*) and characteristics (*b*) for both *Holcocephala* (red) and *Coenosia* (blue). (*a*) Example trajectories (compressed in two-dimensions) of *Holcocephala* (i) and *Coenosia* (ii) are plotted intercepting linear targets, from perch to catch. LoSs are plotted at 50 ms intervals throughout the flight, time resolution of flight is 1 ms. (*b*) From all captured flights, an index of LoS parallelism, range vector correlation (where −1 = parallel navigation), is plotted along the time-normalized flight length for *Holcocephala* (i) and *Coenosia* (ii). Mean points where an optimum course becomes available through acceleration are marked by plotted circles. The angular error between pursuer heading and the optimum course, once one becomes available, is plotted for *Holcocephala* (iii) and *Coenosia* (iv).

Flights of both fly species were reconstructed in 3D (figure 3*a*). Both *Holcocephala* and *Coenosia* use similar mean average flight speeds (0.71 ± s.e. 0.02 ms^−1^ (*n* = 109) and 0.69 ± s.e. 0.02 ms^−1^ (*n* = 59)) and accelerations (mean peak 7.3 ms^−2^ for *Holcocephala* and 9.3 ms^−2^ for *Coenosia*) to intercept targets, even though their mean wingbeat frequencies differ substantially (*Holcocephala* = 130 ± STD 10 Hz (*n* = 10); *Coenosia* = 306 ± STD 19 Hz (*n* = 10)). *Holcocephala* pursues targets at much greater range than *Coenosia* (81–788 mm for *Holcocephala;* 23–212 mm for *Coenosia*). To test for parallel navigation, range vector correlation (an indicator of LoS parallelism), was calculated for both species. *Holcocephala* (figure 3*b*(i)) shows near-parallel navigation (parallel navigation = correlation value −1), with a strong correlation appearing early in the flight (correlation 20% into the flight = −0.8) as already described in [16]. *Coenosia* (figure 3*b*(ii)) also trended towards parallel navigation as the flight progressed, but at a slower rate (correlation at 20%, 50% and 90% of the flight = −0.57, −0.58 and −0.80, respectively). The mean error from optimum heading was 14.1° ± STD 7.4° for *Holcocephala* (figure 3*b*(iii)) and 25.0° ± STD 11.2° (figure 3*b*(iv)) for *Coenosia*. Together, the data indicate that *Holcocephala* has a more optimal controller, or that it may implement turning commands more accurately. Alternatively, the closer range of *Coenosia* flights could simply result in lower performance. Next, we investigated if the pursuer heading rotations within flights are supporting of a pure pursuit, a deviated pursuit or a pro-nav controller. As highlighted by [15], models that predict turning behaviour would be most informative when tested against turning targets. For this reason, both fly species were tested with linear and with erratically moving targets (with changing speed and direction).

### 3.2. Pure pursuit, pro-nav, and deviated pursuit correlations and flight simulations

#### 3.2.1. Pure pursuit test and simulations

**Figure 4.**
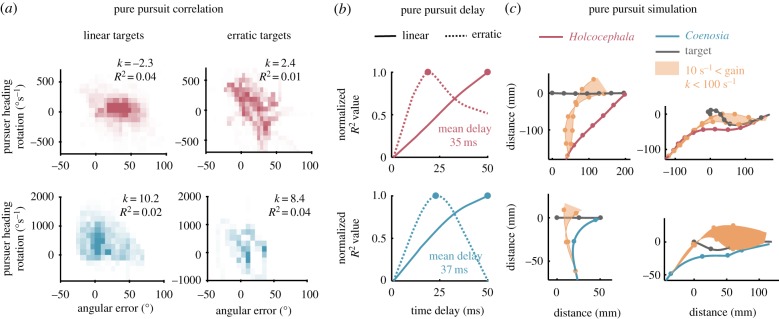
Fitting a model for pure pursuit. (*a*) Purser heading rotation is plotted against the error between pursuer heading and the LoS in both *Holcocephala* (red) and *Coenosia* (blue), shown for linear (left) and erratic (right) targets. Histograms of points are displayed with occupancy representing frequency count within a block. Data are displayed at the time delay that gave the highest coefficients of determination for a linear model. The best fitting model gain (*k*) and resulting coefficient of determination are depicted by each panel. (*b*) Coefficients of determination, normalized from lowest to highest, are plotted against the respective applied time delay between stimulus and recorded response, with peaks marked by points. (*c*) Pure pursuit flight simulations (compressed in two-dimensions) are plotted across a wide range of constant values (trajectory variation represented within the shaded area) and at best fitting delays, using flight speed and target position taken from recorded data. Points mark 50 ms intervals.

A strong positive correlation between pursuer heading angular error from the LoS and rotation rate of the pursuer heading would be expected for a pure pursuit navigation system. However, we did not find such correlation with either linear or erratic targets and for both *Holcocephala* and *Coenosia* (figure 4*a*). The best fitting linear regression models for both flies had little explanatory value (for *Coenosia*, with linear targets *k* = 10.2, *R*^2^ = 0.02 and with erratic targets *k* = 8.4, *R*^2^ = 0.04. For *Holcocephala*, with linear targets *k* = −2.3, *R*^2^ = 0.04 and with erratic targets *k* = 2.4, *R*^2^ = 0.01). For the linear targets, the fit of time constants continually increased to the maximum tested at 50 ms for both species, whereas with the erratic targets this value was 22 ms for *Holcocephala* and 15 ms for *Coenosia* (figure 4*b*). Regardless, the flight simulations with a pure pursuit controller model do not match the trajectories taken by either species of predator (figure 4*c*). In the experiments both fly species steer ahead of the target's position, but in the pursuit simulation the pursuer undershoots the target trajectory and must enter a tail-chase towards the target, only catching it once its linear speed exceeds that of the target. We ran the same simulation with a wide range of constant values (10 to 100 s^−1^), but this did not improve the fit of the model (figure 4*c*). Thus, a pure pursuit controller is not supported by the data.

#### 3.2.2. Pro-nav test and simulations

**Figure 5.**
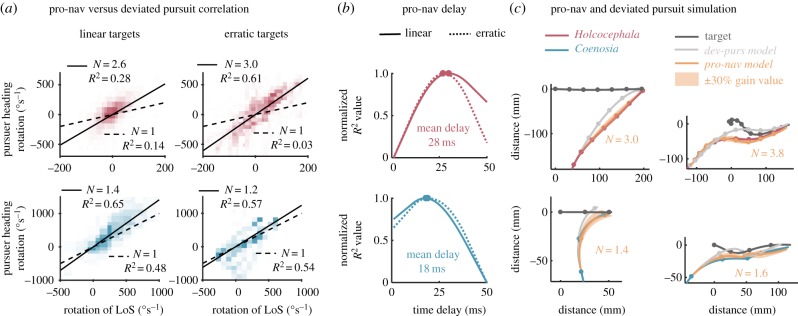
Fitting models for deviated pursuit and proportional navigation. (*a*) Purser heading rotation is plotted against the rotation of the LoS for both *Holcocephala* (red) and *Coenosia* (blue), shown for linear (left) and erratic (right) targets. Histograms of points are displayed with occupancy representing frequency count within a block. Data are displayed at the time delay that gave the highest coefficients of determination for a linear model. The best fitting pro-nav gain constant and resulting coefficient of determination are depicted top left each panel. Deviated pursuit (dev-purs) behaviour, where *N* = 1, is also tested for and the model gain and coefficient of determination displayed bottom-right of each panel. (*b*) Coefficients of determination, normalized from lowest to highest, are plotted against the respective applied time delay between stimulus and recorded response, with peaks marked by points. (*c*) Pro-nav flight simulations (compressed in two-dimensions) are plotted at individually fitted navigation constant values and best-fitting time delays, next to *N* = 1 dev-purs simulations, using flight speed and target position taken from recorded data. Points mark 50 ms intervals.

For interception of targets moving at a constant velocity, we found a correlation between rotation rate of the pursuer heading and rotation rate of LoS, for both *Holcocephala* and *Coenosia*. For *Holcocephala,* the correlation was stronger for the erratic targets. (For linear, *N* = 2.56; *R*^2^ = 0.23, *n* = 109 flights. For erratic, *N* = 3.04, *R*^2^ = 0.59, *n* = 17 flights figure 5*a*.) For *Coenosia*, the strength of the correlation was similar for linear and for erratic targets (*N* = 1.4; *R*^2^ = 0.65, *n* = 59 flights versus *N* = 1.2; *R*^2^ = 0.57, *n* = 8 flights, respectively, figure 5*a*.). This correlation is the hallmark of a proportional navigation control system. These results were obtained with a best-fit temporal delay for linear-erratic targets of 29–27 ms for *Holcocephala* 19–17 ms for *Coenosia* (figure 5*b*). Even though the targets were presented with similar velocities to both species (see Methods section 2.2), for linear targets the mean rotation of the LoS in *Holcocephala* was an order of magnitude less than that of the *Coenosia* (33.4° s^−1^ ± s.e. 0.3° s^−1^ as opposed to 333.8° s^−1^ ± s.e. 3.1° s^−1^). The mean rotation of the LoS in *Holcocephala* for the erratic flights was also less than that of *Coenosia* (60.9° s^−1^ ± s.e. 1.0° s^−1^ as opposed to 228.2° s^−1^ ± s.e. 8.7° s^−1^). For any relative movement of prey normal to the LoS from the reference frame of the predator, the resulting rotation of the LoS is proportional to the inverse tangent of 1/range between target and pursuer. Thus, the higher rotation rates in Killer flies likely arise from the shorter target range on take-off.

The pro-nav steering model results in well-fitted simulated flight trajectories, for both species when intercepting both linear and erratic targets (figure 5*c*), despite not taking account of any potential biomechanical limitations nor environmental perturbations. When the model was tested with sequential fitting of constants incrementing from *N* = 1 to 10, similar peak gain fittings to the correlative measure were found for the navigational constant of both species. For linear targets, the mean best fitting gains were *N* = 3.4 ± s.e. 0.1 (*n* = 109) for *Holcocephala* and *N* = 1.6 ± s.e. 0.1 (*n* = 59) for *Coenosia*. For erratic targets they were *N* = 3.9 ± s.e. 0.1 (*n* = 17) for *Holcocephala* and *N* = 1.4 ± s.e. 0.2 (*n* = 8) for *Coenosia* (electronic supplementary material 3). For linear target intercepts by *Holcocephala*, the simulation showed a mean distance from the true flightpath of 5.8 mm ± s.e. 0.5 mm and angular error of 7.4° ± s.e. 0.6°. For linear target intercepts by *Coenosia*, the mean distance between the simulation and the true flightpath was 3.86 mm ± s.e. 0.5 mm and mean angular error 7.0° ± s.e. 0.6°. For erratic targets intercepts by *Holcocephala*, the simulation had a mean 8.47 mm ± s.e. 1.79 mm distance and 9.7° ± s.e. 1.46° error from the true course. For *Coenosia* intercepts of erratic targets, the simulation had a mean 8.3 mm ± s.e. 2.5 mm distance and 9.0° ± s.e. 1.8° of error from recorded flight paths.

Thus, these results support pro-nav as the underlying feedback controller system.

#### 3.2.3. Deviated pursuit test

A third possibility is that the two predatory species employ a deviated pursuit control controller, which aims to maintain a fixed error angle between pursuer heading and LoS. To maintain a fixed error angle, a rotation in LoS must be exactly matched by a rotation in pursuer heading (1 : 1). Thus, one may suspect a deviated pursuit controller may be in place if the best fit gain constant for a pro-nav controller yields *N* ≈ 1. However, a deviated pursuit controller correlation (*N* = 1) performed poorly when fitted to *Holcocephala* flights towards linear (deviated pursuit *R*^2^ = 0.14) and erratic (deviated pursuit *R*^2^ = 0.03) targets (figure 5*a*) and is therefore unlikely to be the underlying system. For *Coenosia,* the deviated pursuit correlation was also lower for deviated pursuit than for pro-nav, towards both linear (*R*^2^ = 0.48) and erratic (*R*^2^ = 0.54) targets (figure 5*a*), but this difference is not striking, and thus, insufficient on its own to ignore deviated pursuit as the controller for *Coenosia*. A secondary measure of a deviated pursuit controller is not whether it successfully maintains a fixed angle, but whether it turns in proportion to the error from that fixed angle. For this we would expect a linear correlation between the pursuer heading error from the LoS and rotation rate of the pursuer heading to be a positive linear correlation, but with a positive, non-zero intercept. As seen for both *Holcocephala* and *Coenosia* (figure 4*a*) there are no such trends in the data (see pure pursuit test), and on this basis a deviated pursuit controller is unlikely to be driving predatory flights in these species.

### 3.3. Effect of neural delay and proportional gain on performance of flight simulation

**Figure 6.**
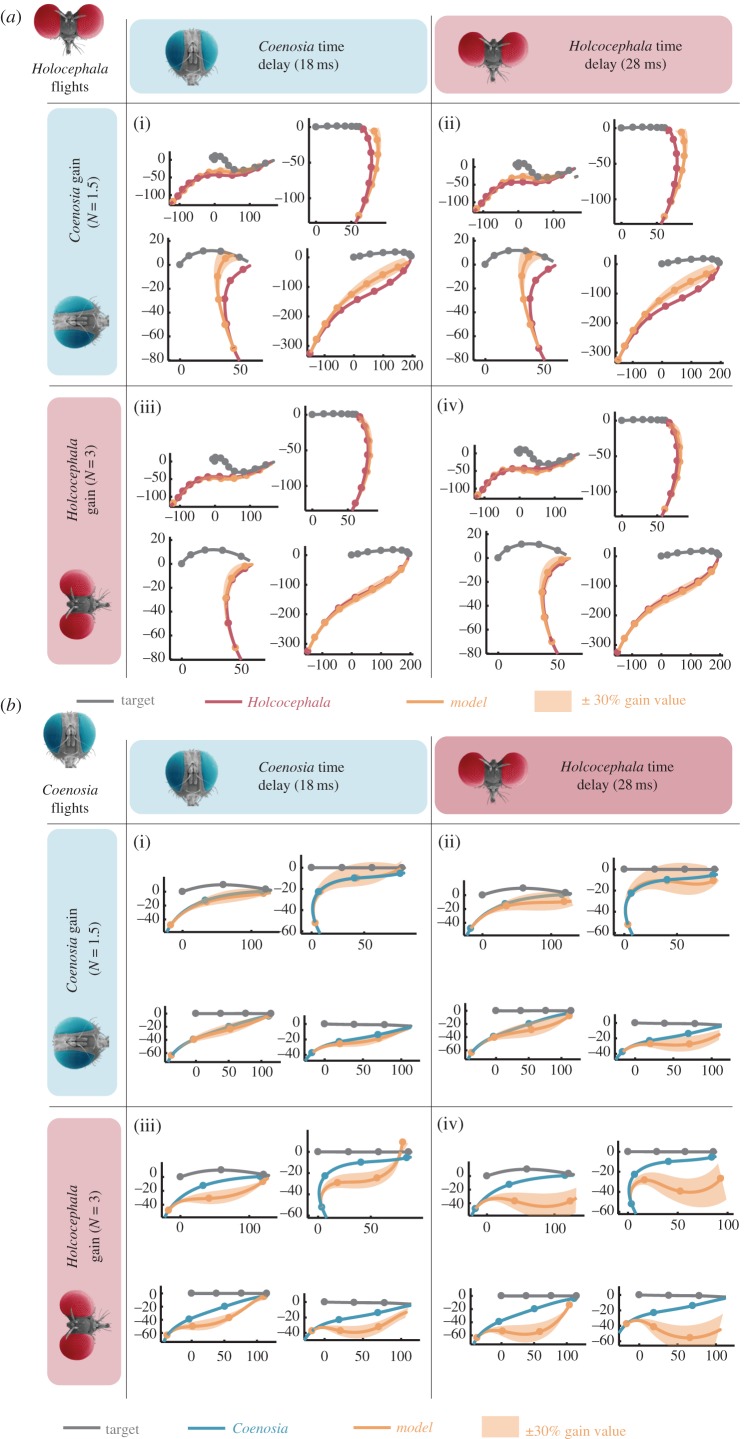
Navigation constant and temporal delay swapping between *Holcocephala fusca* and *Coenosia attenuata.* Best fitting gain and temporal delays are interchanged between both fly species and applied to models overlaid on the four example flightpaths (compressed in two-dimensions). This demonstrates the independent effects of time delay and navigation constant variation on *Holcocephala* (*a*) and *Coenosia* (*b*), under their differing flight parameters such as take-off distance*.* Points mark 50 ms time intervals.

To individually test the effects of differing gain and time delay between the two species, we took four trajectories from each species and ran pro-nav simulations, with the best fit gain and time delay interchanged. Simulating a *Holcocephala* flight with the delay and gain observed in *Coenosia* (*d* = 18 ms and *N* = 1.5; figure 6*a*(i)), results in a longer route to the target. Most of this effect arises from the lower gain (figure 6*a*(ii)), with the shorter delay having little effect (figure 6*a*(iii)). Simulating a *Coenosia* flight with *Holcocephala* delay and gain (*d* = 28 ms and *N* = 3; figure 6*b*(iv)) also results in a longer path to the target. In this case, both, longer time delays (figure 6*b*(ii)) and higher gain (figure 6*b*(iii)) result in additional over-compensating turns by the pursuer and much longer routes to the target.

The effect of the navigation constant on resulting flightpath is dependent on the ratio of pursuer speed to the closing rate between pursuer and target (*V*_p_/*V*_c_ ratio). Both *Holcocephala* and *Coenosia* have *V*_p_/*V*_c_ ratios of near 1. (For linear targets 0.99 ± s.e. 0.02 and 1.08 ± s.e. 0.13 respectively and for erratic targets 1.01 ± s.e. 0.06 and 1.34 ± s.e. 0.60.) Thus, the effective navigational constant *N*′ approximates *N* [22], the importance of this similarity is described in the discussion.

### 3.4. Efficiency of pro-nav versus pure pursuit controller on real flight conditions

**Figure 7.**
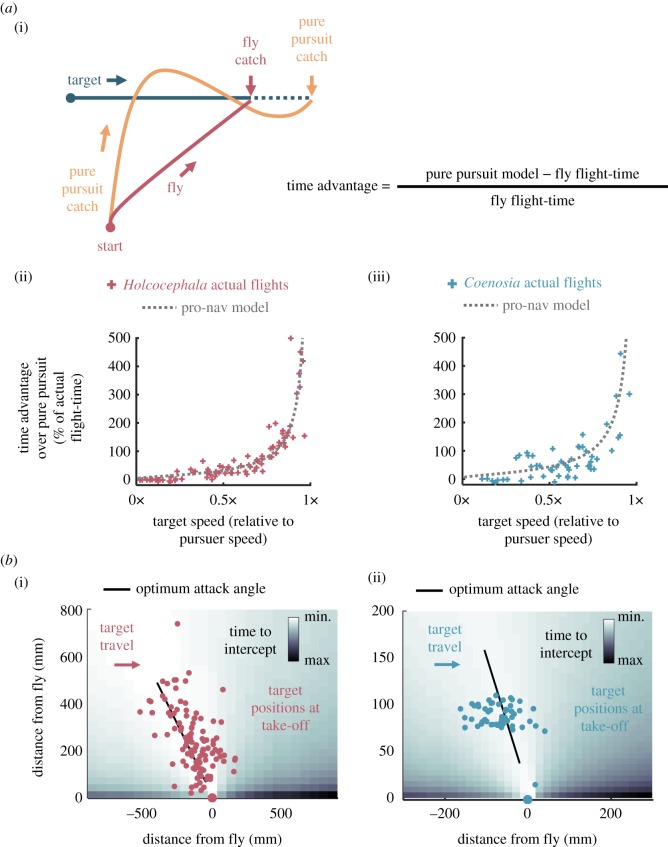
Relative advantages of proportional navigation over pursuit and optimality of take-off positioning. (*a*) (i) Diagrammatic representation of time advantage for flight courses taken by flies compared with a pure pursuit model. Calculated time advantages as a percentage of actual flight-time plotted against the relative speed of the target for (ii) *Holcocephala* (*n* = 109) and (iii) *Coenosia* (*n* = 59). The mean theoretical advantage of a pro-nav model, given the same conditions as the fly is overlaid (dotted line). (*b*) Target positions (relative to pursuer) at the time of fly take-off are plotted for (i) *Holcocephala* (*n* = 109) and (ii) *Coenosia* (*n* = 59). Underneath is a map of relative time-to-contact for different hypothetical target starting positions according to fitted proportional navigation models, travelling at the mean speed of each fly respectively, and launching vertically from the origin. Target headings are aligned to travel left to right across the page.

To quantify the relative efficiencies of investing in proportional navigation over pure pursuit, the relative time-to-target advantage was calculated for both species (figure 7*a*). For this, we used the data from interception of linear targets i.e. by feeding in the starting positions and velocities of both target and predator, then letting the pure pursuit model steer the predator through the simulation flight until contact with target (*Holcocephala n* = 109, *Coenosia n* = 59 flights). We then calculated the flight time difference between the pure pursuit simulation and the real flight. The greater the speed of the target relative to the pursuer, the greater the time advantage of the fly trajectories over a pursuit controller (figure 7*a*(i–iii)). Time-to-contact differences of actual flights versus the pure pursuit model are matched by the time advantages of a theoretical pro-nav controller (with gain matched to the flies, respectively), demonstrating that the change in controller is responsible for time-to-contact savings.

Time-to-contact is also affected by the initial attack angle on take-off (angle between initial LoS and target flight-path). Our flight simulations demonstrate that there is an attack angle which uses minimal time-to-contact for the controller tunings and mean flight-speeds (figure 7*b*). This value is 39° for *Holcocephala* (figure 7*b*(i)) and 35° for *Coenosia* (figure 7*b*(ii)). Both species of fly took off after targets near to this optimum attack angle, with mean attack angles of 32.8° ± s.e. 1.7° for *Holcocephala* and 37.0° ± s.e. 1.9° for *Coenosia*.

## 4. Discussion

The two species in this study see with differing spatial resolutions, but they set out to solve the same challenge, to catch other aerial arthropods. We raised the question as to whether they have evolved the same means of navigating to their target. *Holcocephala fusca* and *Coenosia attenuata* are unlike other studied insect navigational systems [3,4,7,10–12] with the potential exception of dragonflies [6]. This is because they intercept targets without needing knowledge of target properties, implementing pro-nav that turns them towards a near time-optimal course to the target. Given the correlation between the rotation of pursuer heading and LoS, and the agreement of modelled flightpaths with fly trajectories (figure 4 and electronic supplementary material 4), we find the most parsimonious explanation of interception behaviour in both species to be proportional navigation. We firmly rule out pure pursuit navigation, as found in many other studied insect systems, because there is neither correlative evidence nor agreement with model simulation. We also rule out deviated pursuit navigation (which would yield identical behaviour to pro-nav with a constant fixed at *N* = 1), as this explains the correlative data less well than a pro-nav controller and deviated pursuit simulations do not match flight trajectories. Harder to rule out are alternative models that employ rotation of LoS as a measured cue (such as the constant bearing model put forward in humans [17]) and result in flightpaths with similar curves to those obtained from a pro-nav controlled simulation. However, due to its simplicity, pro-nav stands out as still the most parsimonious controller that can explain interception flights as the constant bearing model affects angular acceleration rather than angular velocity and incorporates a damping term. Proportional navigation engenders near identical results with fewer input variables and is simpler to implement, and thus we suggest much more likely.

Correlative and simulation fitting methods produced consistent results: *Holcocephala* appear to use a control gain of *N* ≈3 to steer their flightpath. This value matches the lower end of the envelope of gain constants used in human mechanisations of pro-nav, such as in guided missiles (3 ≤ *N* ≤ 5) [22]. Additionally, the terminal attack phase of the peregrine falcon, *Falco peregrinus*, is modelled well by proportional navigation with mean gain of *N* = 2.6 [15], which is similar to the gain found in *Holcocephala*. The efficacy of the navigation constant is also dependent on target motion, and to describe this, the effective navigation constant (*N*′) is used, where:
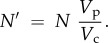


*V*_p_ being the predator speed and *V*_c_ the closing speed between pursuer and target. For intercepting a non-manoeuvring target, the control optimum *N*′ is 3, ensuring zero-miss with minimal cost of control (integral square of lateral accelerations) (for detailed work-through see [22,26]). For near-stationary targets the *V*_p_/*V*_c_ ratio is 1 but for intercepting moving targets, as with the two flies in this investigation, *N*′ may potentially vary from *N*. We found that for both flies *N*′ ≈ *N* and thus *Holcocephala* uses near the optimum control gain of *N*′ = 3. *Coenosia* use a low gain of near *N* = 1.5. This is below the optimal control level and below the range used in missiles [22]. This could represent limitations given by the high LoS rates they experience through close target proximity and the necessary neural lag between stimulus and reaction. We demonstrate in figure 6 that given the proximity to target at which *Coenosia* hunt, employing an optimal control gain or longer time delay would frequently cause overcompensation in the turning response, and thus longer paths to the target. *Coenosia* maintains a course further from optimal than *Holcocephala* does. By only studying flight traces and range vector correlation, it would be easy to conclude that that they are not attempting to fulfil parallel navigation, as previously reported for dragonflies [18]. However, simply because killer flies do not successfully maintain near parallel LoS, it does not mean that they do not use pro-nav, as demonstrated by the model's accurate description of their behaviour.

We suggest that the difference in gain intimately reflects the differences in physiology and predation tactics of the two species. The higher acuity of *Holcocephala* vision enables them to spot suitable targets at greater range [16], and thus encounter lower LoS rates and use the optimal control gain to steer into targets. The lower acuity vision of *Coenosia* [21] results in close proximity attacks that create high rotations in LoS and necessitate a short time delay and lower gain. Most significantly, this study can be compared with the only other described use of proportional navigation in an animal, that of the peregrine falcons [15]. It is remarkable that peregrines, operating at much greater speeds and with radically different flight morphology use the same system as miniature predatory flies, and with a very similar near-optimal gain tuning to that of *Holcocephala*. This demonstrates that proportional navigation could well underlie interception behaviours across further animal taxa (e.g. [6,13,14]). Moreover, the comparison between *Holcocephala* and *Coenosia* needs to be augmented by further species that are physiologically dissimilar and hunt in differing habitats. Such studies would give evidence to explanations for the distinct control gains or present diverse specializations of the control system to reflect the variation between differing groups' tasks, physiology or geometry of interaction.

An alternative explanation of the lowered gain of *Coenosia* involves the parasitic attitude loop. This effect should be familiar to biologists in principle, although not by this name. To rotate its heading, the fly must rotate its body. This rotation potentially affects the measurement of LoS rate of the target and could create instabilities in tracking. These can be prevented by reducing the navigation constant [27]. It is unlikely that the parasitic attitude loop is responsible for the gain differences found in this study, as flies (like other animals) are most likely capable of accounting for rotations of their own bodies to stabilize vision [18,28,29]. This accounting can either be conducted predictively [18,30] or reactively [31,32] to separate self-induced rotation of the pursuer body from actual rotation of the LoS relative to the exocentric reference frame.

Correlative evidence for pro-nav in *Holcocephala* was weakest for interceptions of linearly travelling targets. These flights had relatively low LoS rates, meaning any noise in tracked positions of target and fly will more easily mask the effects of the control system, thus resulting in a lower sensitivity to constant fitting. When LoS rates were increased by using erratically travelling targets, the correlation of LoS rate and pursuer turning rate of *Holcocephala* showed stronger evidence for a pro-nav controller. The anatomy of *Holcocephala* suggests some potential clues to their implementation of pro-nav. Their highly specialized fovea seems likely to track the target, resulting in rotation of the head relative to the body as in the gimbal seeker of a missile. By maintaining target fixation and using either visual or inertial cues for rotation of the head, the fly can measure rotations in the LoS relative to the exocentric reference frame and thus conduct pro-nav. The use of pro-nav does not exclude the possibility that internal models are used to maintain gaze fixation on the target, reducing tracking lag as found in dragonflies [18] (likely through a corollary discharge), only that the rotation of fixated gaze is fed into the pro-nav controller.

Thus, we raise the question, not why both *Holcocephala* and *Coenosia* use a proportional navigational controller to attack targets, but why other described species of fly use a pursuit controller? Proportional navigation is likely to have a higher cost of implementation than a pursuit controller, or a narrower applicability. Most of the work hitherto completed on dipteran aerial tracking used the approach to conspecific targets (e.g. [4,11,12]). This is a fundamentally different problem than the one faced by a predator. While predators are subject to strong selective pressure to successfully grapple with targets, social engagements involving pursuit may not be selected for success of aerial interception, instead simply following a potential mate or chasing away a rival may count as a ‘success’ without need for actual contact. In cases of conspecific interception, innate knowledge of the target allows for biasing of any potential control system given that assumptions can be made about rough flight speeds and target sizes. Just such biasing underpins initiation of aerial interception of female hoverflies by males [3]. We have discounted sexual motivations in flights for both species; female *Coenosia* chase all conspecifics with cannibalistic purpose (males need to out manoeuvre females to initiate mating) and male *Holcocephala* search for stationary (perched) females before attempting to copulate. It is therefore unlikely that the recorded behaviour towards moving dummy targets was other than predatory in function.

The relative simplicity of aligning axis of motion directly along the LoS to the target may have lower physiological and computational requirements than the variable coupling of thrust axis and LoS required for proportional navigation. To investigate further, knowledge of the head–body relationship during flight would be required, which is challenging for the two species studied in this work due to their small size. Robber flies (Asilidae) are a large family of flies, containing genera with much greater body size than *Holcocephala* (i.e. *Microstylum* or *Laphria*). These groups would make head–body relationships a more tractable question, should they also use pro-nav to intercept targets. This is our current line of work, assisted by electrophysiological work into the relationship between target stimuli and the motor commands transported to steering muscles down the descending neurons. However, robber flies are a large family of flies with extremely diverse hunting methods. Many are not sit-and-wait predators like *Holcocephala*, instead actively foraging for prey [33]. The results of this study with *Holcocephala fusca* should not be taken to transfer across to other species of robber fly. Just as the different hunting habits of *Coenosia* and *Holcocephala* have resulted in different gains on their control systems, the difference in other robber fly hunting styles may mean their target-navigation systems are entirely different.

Both predatory fly species here studied, *Coenosia attenuata* and *Holcocephala fusca*, take-off while targets are in the time-optimum catch window. This does not necessarily suggest that flies wait for targets to reach this window, but that they might simply apply a filter to their target selection preferences or align their body orientation for this purpose. For instance, *Holcocephala* most often sit with their body at 30–50° in elevation, potentially aligning their visual axis along the optimum take-off window. If most targets are likely to be flying roughly parallel to the ground, a fly then need only give preference to targets coming towards it, with the time-optimum point for take-off coinciding with the target moving into the centre of its visual field. Additional work is needed to elucidate the exact cues that trigger the predatory behaviour at a particular time for both species, which would demonstrate how these animals take-off while targets are in the optimum catch window.

## 5. Conclusion

By studying the behaviour of the small flies *Holcocephala fusca* and *Coenosia attenuata*, we demonstrate that intercepting prey by flying a near time-optimal course need not be underpinned by forward prediction of target path. Instead, the behaviour of both species is explained by proportional navigation, the basis of predatory behaviour in peregrine falcons and underpinning guidance in modern missiles. *Holcocephala* uses a higher, optimal gain of near *N* ≈ 3 to steer into targets over long ranges, similar to findings in falcons. In contrast, *Coenosia* uses a lower gain of near *N* ≈ 1.5, potentially overcoming high rotations in LoS created by their proximity to targets at take-off. Both species have short time delays on their control systems, *Holcocephala* at 28 ms and *Coenosia* at 18 ms. The simplicity of implementation and energetic savings of proportional navigation mean that there is a wide applicability and suggest it may underpin predatory interception in other organisms. Studying such biological implementations may improve our general understanding as the organisms studied may demonstrate energetic saving tactics and such innovations could lead to improved human produced proportional navigation controlled systems.

## Supplementary Material

Parallel Navigation, Proportional Navigation and Range Vector Correlation

## Supplementary Material

Flattening to the Engagement Plane

## Supplementary Material

Simulation Constant Fitting

## Supplementary Material

Curve Fittings

## Data accessibility

The data used for this manuscript (digitized flight trajectories) can be found in the Dryad Digital Repository (https://dx.doi.org/10.5061/dryad.2jc05rt).
