# Supplementary material for: Interception by two predatory fly species is explained by a proportional navigation feedback controller
Source: J R Soc Interface. 2018 Oct 17;15(147):20180466. doi: 10.1098/rsif.2018.0466 (PMC6228472; doi:10.1098/rsif.2018.0466)
Supplement: Parallel Navigation, Proportional Navigation and Range Vector Correlation [file rsif20180466supp1.pdf]

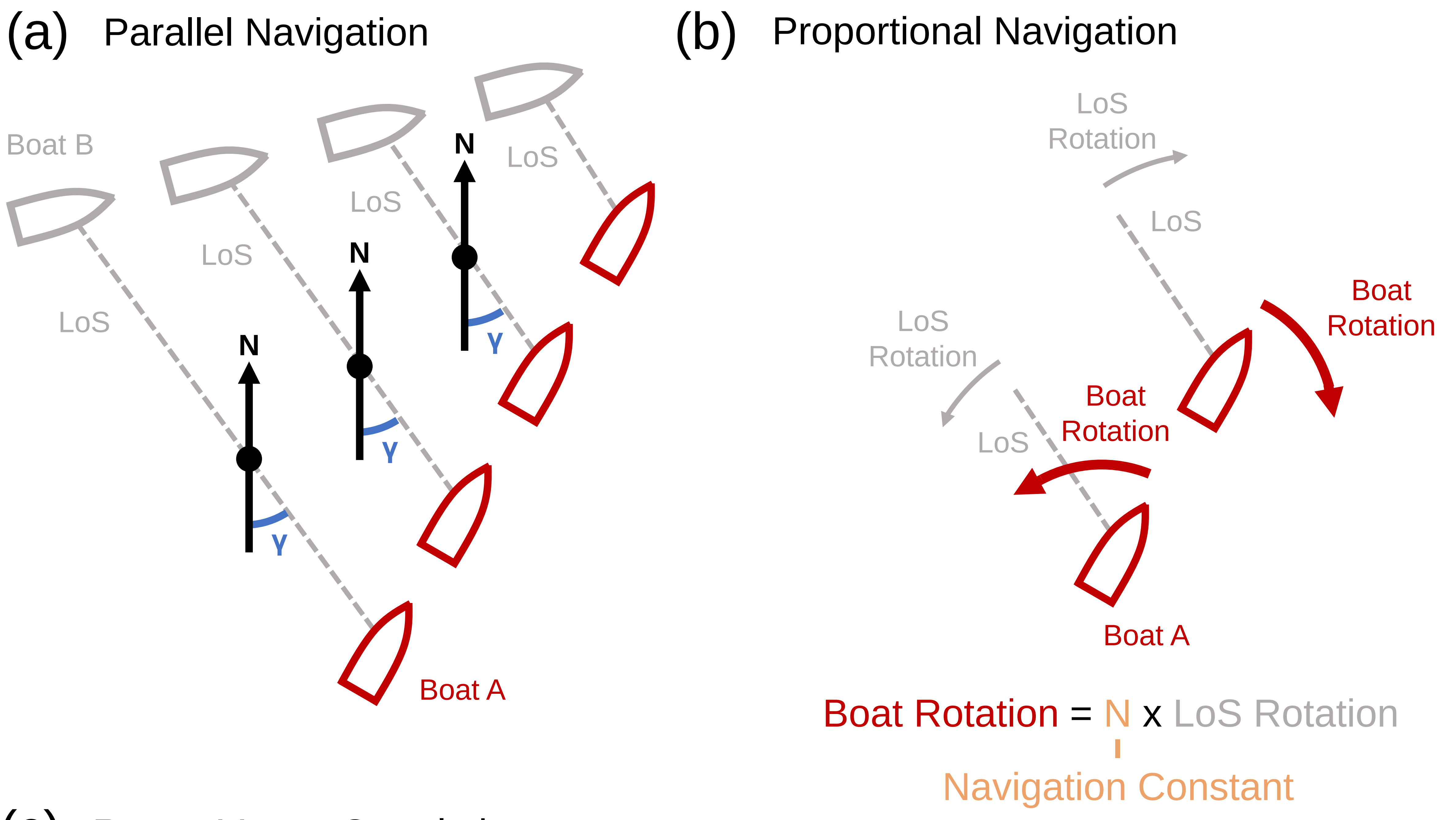

(c) Range Vector Correlation

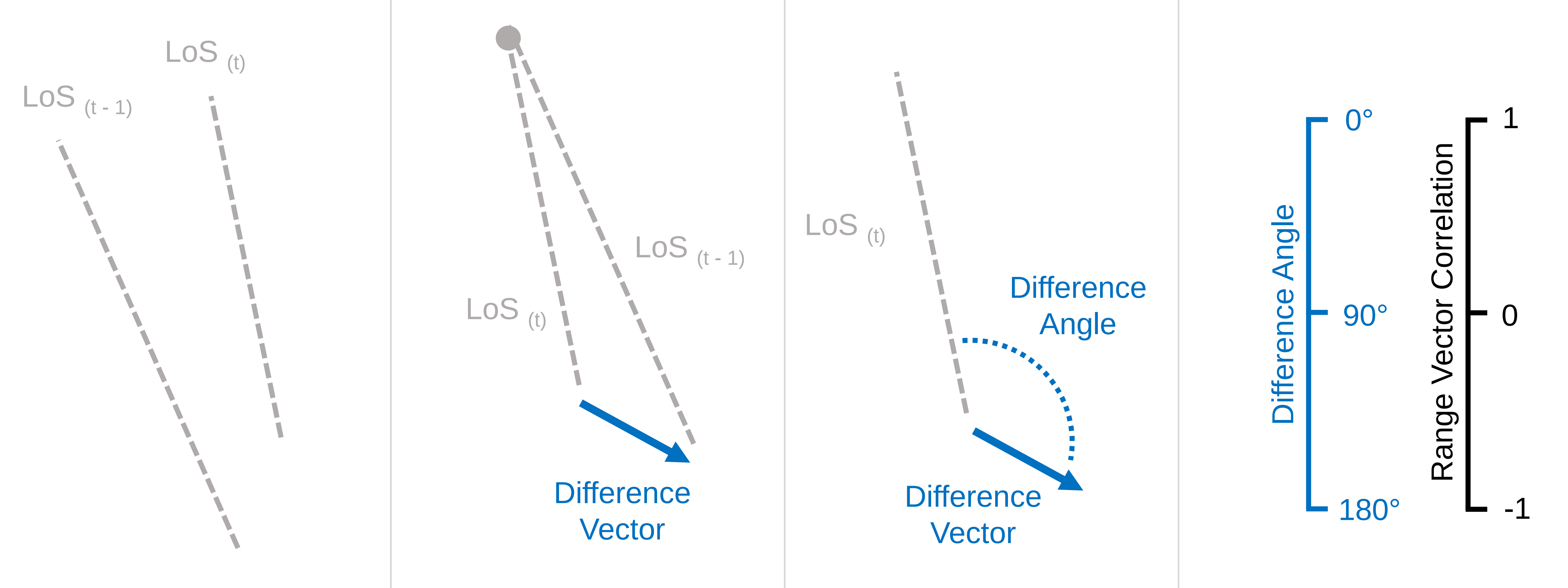

Step 1	Step 2	Step 3	Step 4
Obtain lines-of-sight between target and interceptor.	Obtain the time derivative difference vectors of LoS.	Measure the angle between difference vectors and LoS.	Convert the difference angle to correlation value.
