# Supplementary figures and images for: Interception by two predatory fly species is explained by a proportional navigation feedback controller
Source: J R Soc Interface. 2018 Oct 17;15(147):20180466. doi: 10.1098/rsif.2018.0466 (PMC6228472; doi:10.1098/rsif.2018.0466)

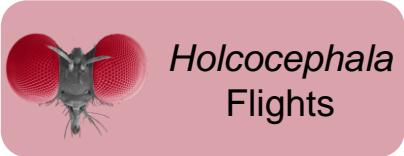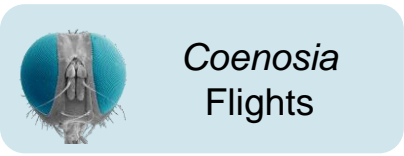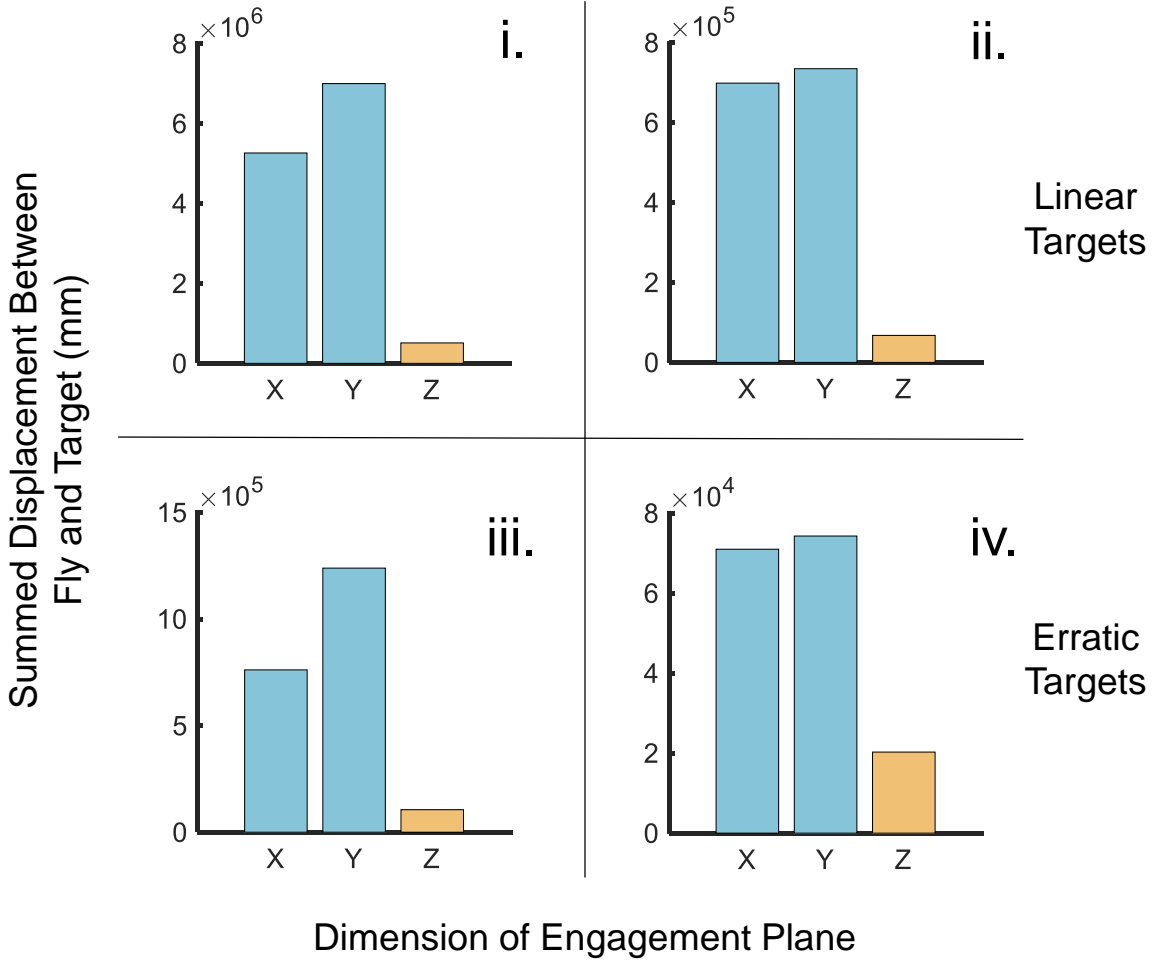

**Supplement 2.**

Supplement: Flattening to the Engagement Plane [file rsif20180466supp2.pdf]

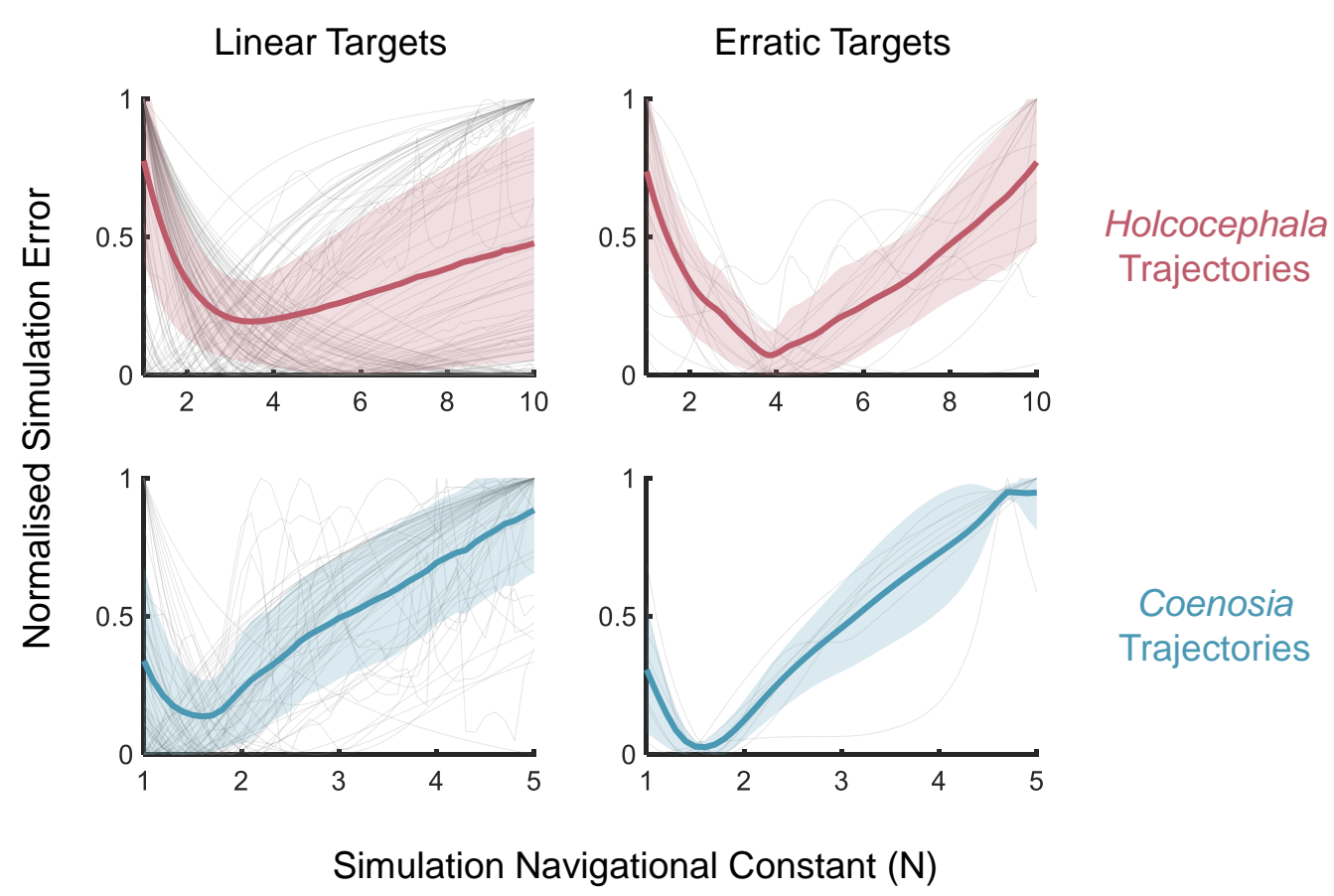

**Supplement 3.**

Supplement: Simulation Constant Fitting [file rsif20180466supp3.pdf]

(a) *Holcocephala fusca*

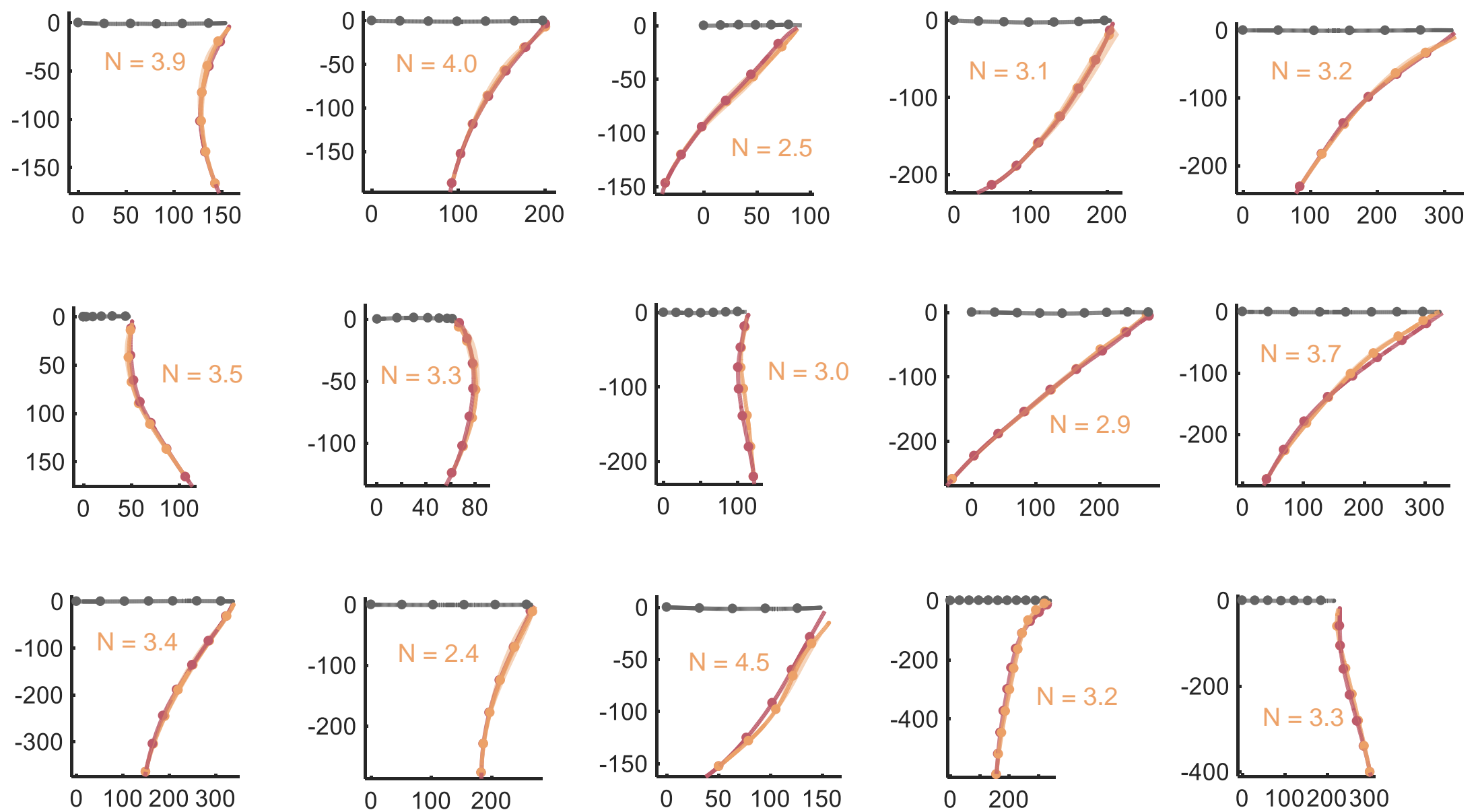

(b) *Coenosia attenuata*

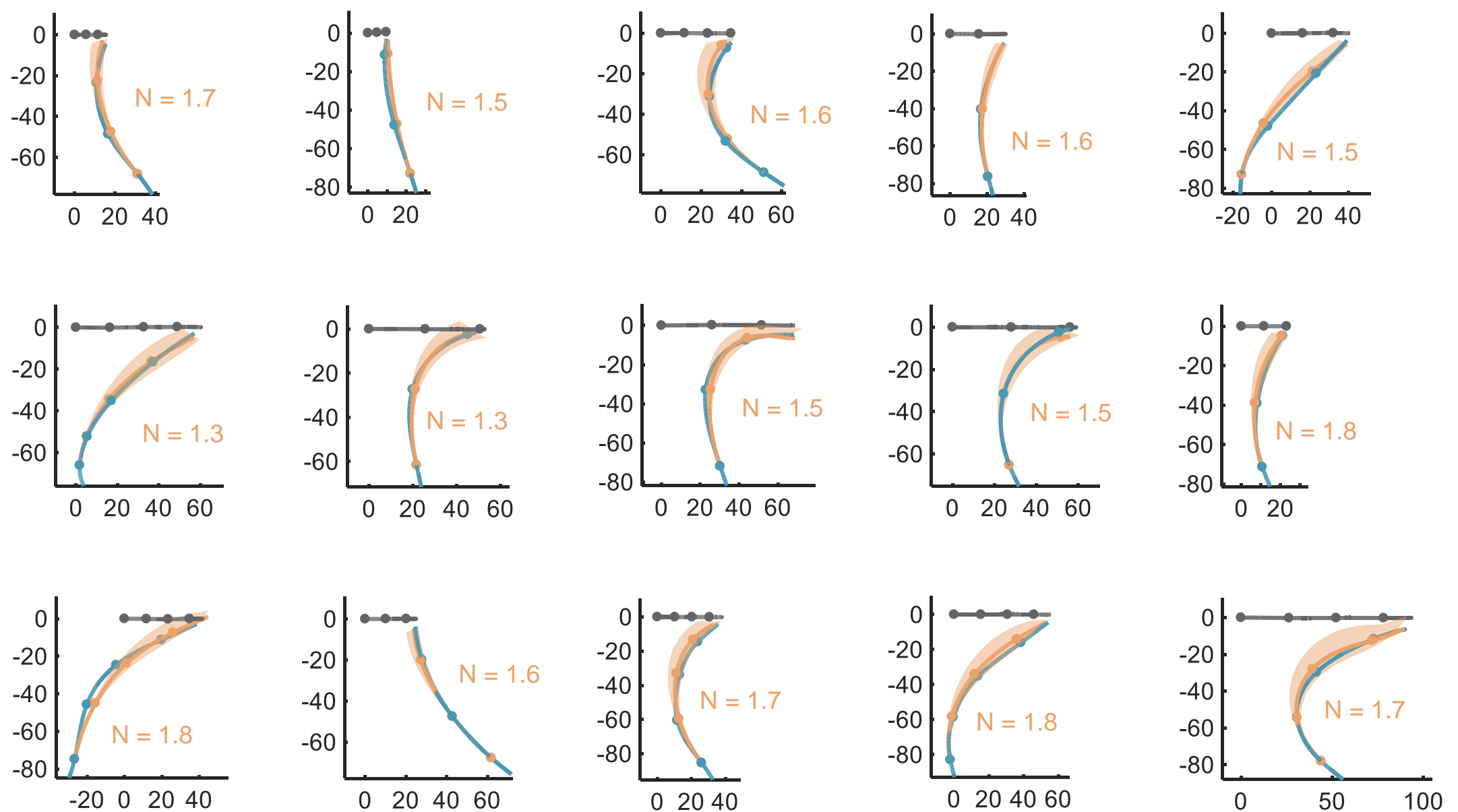

Supplement 4.

Supplement: Curve Fittings [file rsif20180466supp4.pdf]
